# The knowledge structure and research trends between light and myopia: A bibliometric analysis from 1981 to 2024

**DOI:** 10.1097/MD.0000000000038157

**Published:** 2024-05-17

**Authors:** Shuaibing Zhou, Yueyue Niu, Xuejiao Li, Juan Yue, Hongmin Zhang

**Affiliations:** a Zhengzhou University People’s Hospital, Henan Provincial People’s Hospital, Henan Eye Hospital, Henan Eye Institution, Henan Key Laboratory for Ophthalmology and Visual Science, Zhengzhou University, Zhengzhou, China; b Henan University People’s Hospital, Henan Eye Hospital, Zhengzhou, China; c Department of Ophthalmology, Sanmenxia Central Hospital, Sanmenxia, China.

**Keywords:** bibliometrics, emmetropization, light, luminance, myopia, wavelength

## Abstract

**Background:**

This bibliometric analysis explored the knowledge structure of and research trends in the relationship between light and myopia.

**Methods:**

Relevant literature published from 1981 to 2024 was collected from the Web of Science Core Collection database. Visual maps were generated using CiteSpace and VOSviewer. We analyzed the included studies in terms of the annual publication count, countries, institutional affiliations, prolific authors, source journals, top 10 most cited articles, keyword co-occurrence, and cocitations.

**Results:**

A total of 525 papers examining the relationship between light and myopia published between 1981 and 2024 were collected. The United States ranked first in terms of the number of publications and actively engaged in international cooperation with other countries. The New England College of Optometry, which is located in the United States, was the most active institution and ranked first in terms of the number of publications. Schaeffel Frank was the most prolific author. The most active journal in the field was *Investigative Ophthalmology & Visual Science*. The most frequently cited paper in the included studies was written by Saw, SM and was published in 2002. The most common keywords in basic research included “refractive error,” “longitudinal chromatic aberration,” and “compensation.” The most common keywords in clinical research mainly included “light exposure,” “school,” and “outdoor activity.” The current research hotspots in this field are “progression,” “refractive development,” and “light exposure.” The cocitation analysis generated 17 clusters.

**Conclusion:**

This study is the first to use bibliometric methods to analyze existing research on the relationship between light and myopia. In recent years, the intensity and wavelength of light have become research hotspots in the field. Further research on light of different intensities and wavelengths may provide new perspectives in the future for designing more effective treatments and interventions to reduce the incidence of myopia.

## 1. Introduction

Myopia is the most common type of refractive error and is one of the most common eye diseases in the world. The prevalence of myopia has increased in recent decades, and some academics have even described it as an “epidemic,”^[1–3]^ with prevalence rates ranging from 10% to 30% among adults in many countries. The prevalence rate among young individuals with higher education (12–13 years of education) is as high as 70% to 90% in several regions of East Asia and Southeast Asia.^[4,5]^ Childhood myopia is likely to progress to high myopia in adulthood. High myopia lengthens the axial length, and axial elongation thins the retina, which can cause major issues and irreversible vision loss, such as retinal tears, retinal detachment, glaucoma, myopic macular degeneration, optic neuropathy, cataracts, and choroidal neovascularization.^[6]^ Therefore, reducing the incidence of myopia is an urgent and primary public health concern that needs to be considered.

Myopia control and prevention are major concerns. Currently, myopia is prevented and controlled by a variety of methods, among which behavioral interventions (such as outdoor activities and outdoor lighting) are widely discussed due to their cost-effectiveness and ease of implementation. As Chakraborty pointed out,^[7]^ recent research has resurrected the 19th-century theory that children’s exposure to light may impact their refractive development. Numerous studies have shown that outdoor light exposure is associated with the incidence and prevalence of myopia,^[8–10]^ and outdoor light exposure can slow the progression of myopia.^[11]^ In addition to the association between outdoor natural light and myopia, different wavelengths of light have varying effects on myopia. Low-intensity long-wavelength red light has been shown to slow the progression of myopia, according to a cohort study by Zhou et al in eastern China.^[12]^ However, there are currently many different perspectives on the relationship between light and myopia; therefore, it is necessary to review the literature in this field.

Bibliometrics is a quantitative research methodology based on text data, publications, and citations. It comprehensively evaluates the dynamics and advancements within a subject or scientific field by categorizing, collecting, analyzing, and mining a substantial amount of scientific data.^[13–15]^ Detailed information about authors, keywords, journals, countries, institutions, and references is obtained during the analysis. Bibliometrics commonly utilizes visualization software to present results. Visualization facilitates the exploration of complex patterns more smoothly and vividly than traditional scientific research methods.^[16,17]^

Due to the growing number of articles on the relationship between light and myopia in recent years, it is difficult for scholars to perform a literature review encompassing the breadth of related research papers. Traditional literature reviews may no longer yield a comprehensive understanding of a research field.^[18]^ However, a substantial amount of scientific data can be analyzed via bibliometric analysis,^[19]^ which has been utilized in many distinct fields and is becoming increasingly popular among academics.^[20,21]^ This study employed bibliometric analysis to provide valuable insights for scholars pursuing knowledge advancement in this field by conducting descriptive statistics and visual analysis of the literature in this area over the past 40 years.

## 2. Materials and methods

### 2.1. Data source and search strategy

To guarantee the accuracy of the data, the Web of Science Core Collection was chosen as the data source for this study, and the index chosen was SCI-EXPANDED. The Web of Science is a high-quality digital database of scientific literature, and it is viewed by many academics as the most appropriate database for bibliometric analysis since it includes articles from a wide range of fields.^[22,23]^ Two authors (S.B.Z. and Y.Y.N.) participated in the data retrieval and extraction process and engaged in further discussions and verification of discrepant literature to minimize potential data bias introduced by human factors. The search strategy was as follows: (TS = (myopi* OR nearsighted* OR shortsighted* OR emmetropization OR ametropia OR “refractive development” OR “refractive stat*” OR “refractive error*” OR “eye growth”)) AND (TS = (light* OR luminance OR illumina* OR wavelength OR *chromatic)). The time span was 1981 through 2024. The literature search was completed on March 10, 2024, the publication language was English, and any kind of literature was allowed. A total of 2769 articles were retrieved. Articles that deviated too far from the topic were discarded to confirm the accuracy of the data according to the title, abstract, and keywords. The final step involved the exclusion of duplicates using the duplication removal function of CiteSpace, yielding a sample size of 525 articles. The retrieved data were stored in “plain text file” format with “full records and cited references”, and basic data on each article, including the author, journal, country, organization, keywords, title, abstract, and references, were extracted. Table 1 shows a summary of the data sources.

**Table 1:** Summary of data source and selection.

Category	Standard requirements
Research database	Web of Science core collection
Citation indexes	SCI
Searching period	January 1981 to January 2024
Language	English
Searching strategy	(TS = (myopi* OR nearsighted* OR shortsighted* OR emmetropization OR ametropia OR “refractive development” OR “refractive stat*” OR “refractive error*” OR “eye growth”)) AND (TS = (light* OR luminance OR illumina* OR wavelength OR *chromatic))
Document types	Unlimited
Data extraction	Export with full records and cited references in plain text format
Sample size	525

### 2.2. Methodology

This study employed CiteSpace (version 6.3. R1, Drexel University, the United States) and VOSviewer (version 1.6.18, Centre for Science and Technology Studies, Leiden University, the Netherlands) to generate visual maps. CiteSpace and VOSviewer are the two most frequently applied bibliometric mapping software programs in this sector.^[24]^ CiteSpace is a citation analysis visualization software developed in 2004 by Professor Chaomei Chen in the context of scientometrics and data visualization. It is currently among the most widely used bibliometric analysis programs.^[25]^ Through time-variant mapping from the research front to the intellectual base, CiteSpace explores the dynamic evolution of a subject.^[25]^ Using CiteSpace, we calculated the centrality of the institution, which measures the importance of the node’s position in the network.^[25]^ VOSviewer is a free software for creating and displaying bibliometric maps created by Van Eck and Waltman. It uses a probability-based approach to data standardization and offers a visual atlas in the areas of keywords, co-organization, coauthor, etc. Convenience mapping and generation of a straightforward, concise visual are among its features.^[26]^ VOSviewer’s clustering function allows clustering of keywords, and different colors represent different clusters. Clustering is a term used in bibliometrics, and keyword clustering is used to identify themes, as keywords frequently occurring together are thematically related.^[27]^ These two programs complement each other, and each has unique characteristics. This study analyzed the knowledge structure of this subject and evaluated the contribution of various research components to this field by examining the research constituents of the 525 studies gathered, such as countries, institutions, journals, and authors.

### 2.3. Data preprocessing

The retrieved data was preprocessed. As some authors’ names appeared in several forms, these forms were amalgamated, and only one form was chosen to increase the accuracy of the statistical data. Furthermore, the number of core authors in this field was calculated using Price’s Law with the following formula: ***m*** represents the minimum number of core author publications in a given field and $n_{max}$ represents the number of papers with the most productive authors in the field.^[28]^


$$m=\text{0}.\text{749}\times \sqrt{n_{max}}$$

**Figure 1.**
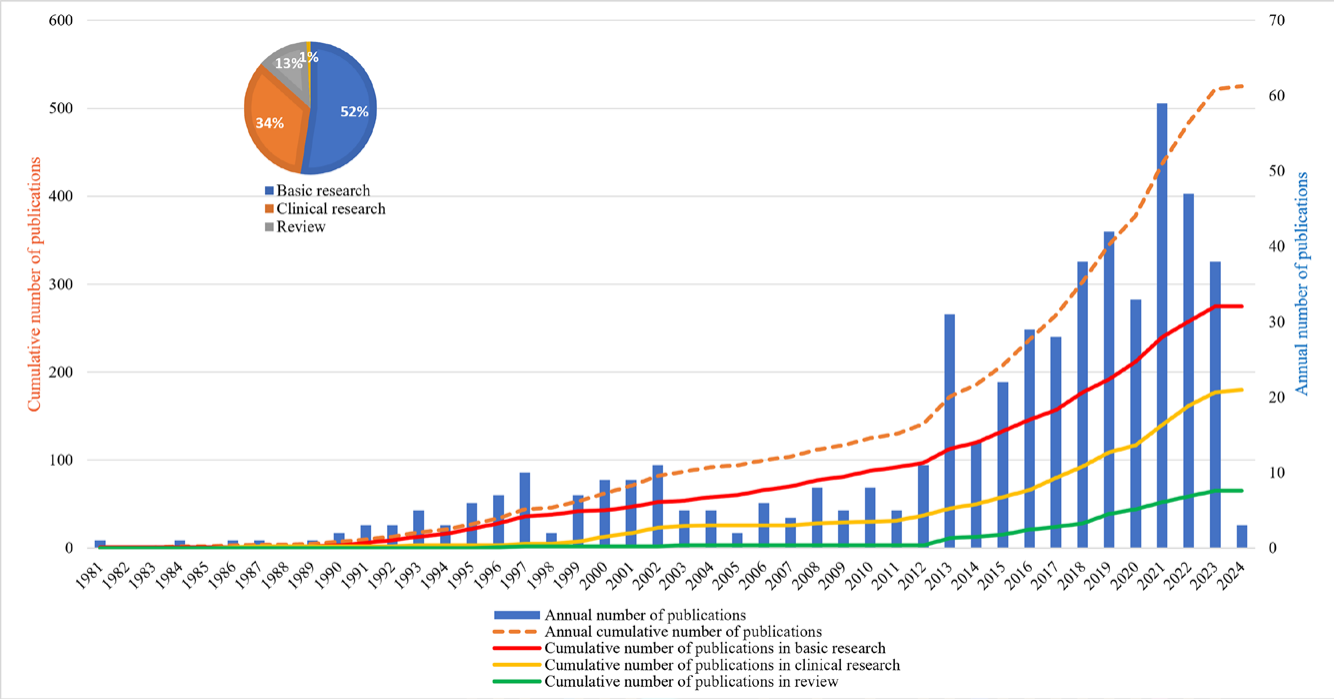
Publication trends from 1981 to 2024 on the relationship between light and myopia. The pie chart in the top left corner illustrates the distribution of research types and their respective proportions.

England, Scotland, Northern Ireland, and Wales were combined into the United Kingdom, and Taiwan was grouped as part of China. Keyword synonyms were combined into groups. Considering that keywords were not used in the literature published before 1990, the period of 1990 to 2024 was selected for keyword analysis. The time slice was set to the default option for all visualizations created by CiteSpace software (1 year). To better explore the structure of this research field, the 525 included studies were split into 4 categories based on study type: basic research (n = 275), clinical research (n = 180), reviews (n = 65), and others (n = 5). Since there were only 5 other articles, this article primarily focused on basic and clinical research as well as reviews. The scale maps for each type of study are displayed in Figure 1.

## 3. Results

The 525 papers included in this analysis were written by 1474 authors from 434 organizations in 37 countries, published in 107 journals, and cited 8665 references from 1910 journals.

### 3.1. Distribution of publications

This study included a total of 525 articles. The time trend chart of published articles on the relationship between light and myopia is shown in Figure 1. The annual cumulative number of published articles showed that the number of papers published in this scientific field has increased over time. The publication trends were separated into 2 stages based on the overall volume of publications in the field. A total of 141 papers were published in the first stage (1981–2012), which was a stable development period and accounted for approximately 27% of all publications. Fewer than 10 papers were published yearly during this stage, a relatively low quantity. The second stage was a rapid development stage (2013–2024), during which the number of articles published gradually increased, and the research area started to attract the attention of an increasing number of academics. A total of 384 papers, or 73% of all papers, were published during the rapid development stage. The annual publication output reached a peak of 59 articles, which was nearly 6 times higher than the number of publications during the stable development stage. Regarding research type, basic research was conducted during the earlier years. The earliest paper on this topic was published in 1981. The volume of published basic research articles has always been the highest, while clinical research and review papers appeared later. However, the quantity of clinical research articles published rapidly increased during the rapid development stage.

### 3.2. Analysis of countries

**Figure 2.**
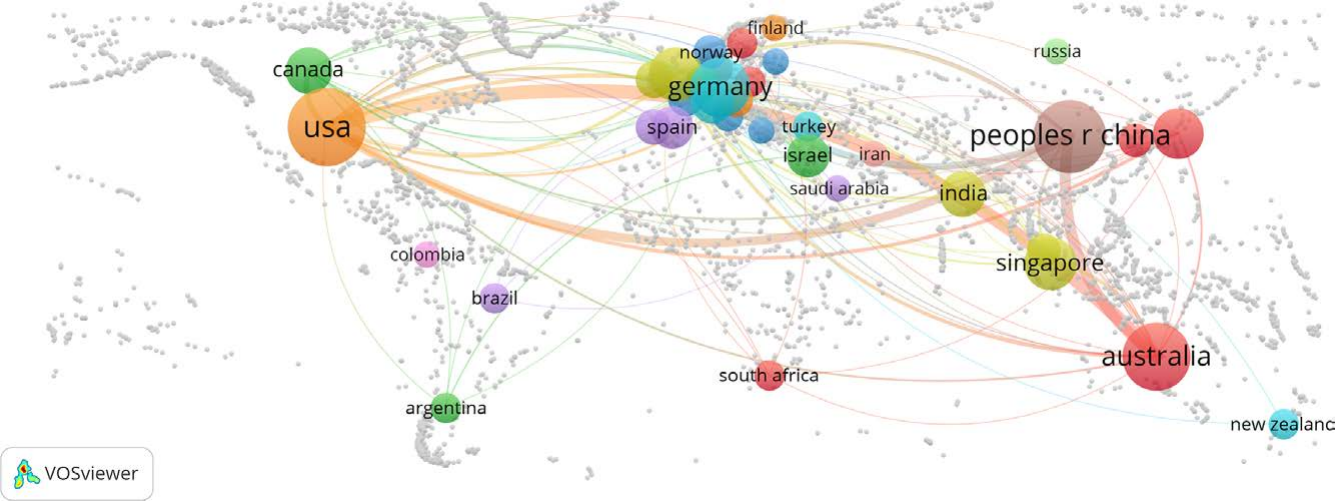
International research cooperation on the relationship between light and myopia. The number of articles published by a country is indicated by the size of the circular node; the larger the circle node is, the more articles the country has published. Lines connecting the nodes show the strength of the association; the thicker the lines are, the more frequently 2 countries cooperate. USA = The United States.

A total of 37 countries have published papers on the relationship between light and myopia. Table 2 lists the top 10 countries with the most articles published among these 37 countries. As shown in Table 2, the United States has produced the most research papers in this field (206 papers published), accounting for 39.2% of the total number of papers published, with 4963 citations and 24.1 citations per paper. China and Australia were the second- and third-largest contributors to this field, with 142 and 98 papers, respectively. The former garnered a total of 2707 citations, while the latter received 3233 citations. The numbers of citations per paper for China and Australia were 19.1 and 33.0, respectively. Approximately 85.0% of all the papers were published in China, Australia, and the United States. In other words, few countries have produced the majority of the publications in this field. Singapore had the highest number of citations per paper, with 28 papers receiving 1286 citations (45.9 citations per paper). Figure 2 shows the collaborative relationships among different countries. As shown in Figure 2, of the top 3 countries in terms of publication volume, the United States has actively collaborated with other countries. China and Australia have also engaged in substantial international collaboration.

**Table 2:** The top 10 countries with the highest number of publications.

Rank	Country	Number of publications	Number of citations	Average citation/publication
1	USA	206	4963	24.1
2	China	142	2707	19.1
3	Australia	98	3233	33.0
4	Germany	53	1973	37.2
5	United Kingdom	30	920	30.7
6	Singapore	28	1286	45.9
7	Japan	24	384	16.0
8	Canada	15	366	24.4
9	India	14	53	3.8
10	Switzerland	11	48	4.4

### 3.3. Analysis of institutions

**Figure 3.**
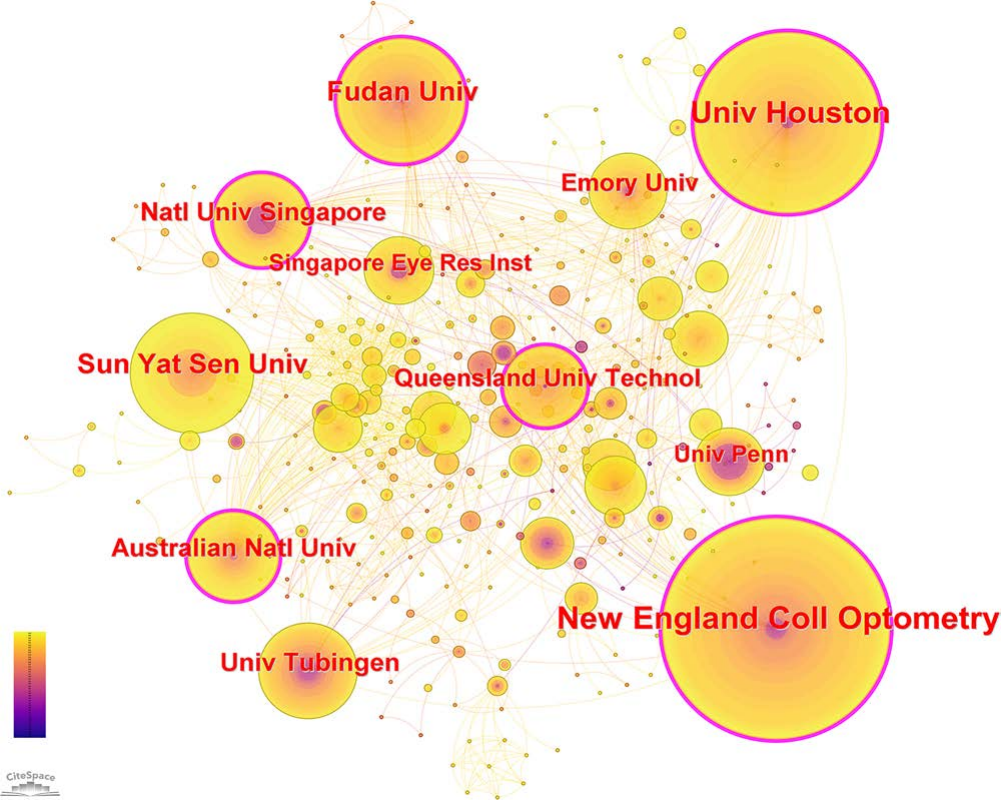
Institution research collaboration networks on the relationship between light and myopia. The size of the node indicates the number of papers published by the institution, and the color variation within the node indicates the year; the darker the color is, the older the paper. Nodes with centrality values > 0.1 are represented by purple circles, and these nodes are considered critical nodes.

A total of 434 institutions have published papers in this area. The top 10 institutions with the most publications are listed in Table 3. The New England College of Optometry in the United States produced 55 articles, the highest number of publications among all the institutions. The University of Pennsylvania had the most citations per publication, with 57.6 citations per publication, followed by the National University of Singapore, with 52.7 citations per publication. The University of Tübingen in Germany ranked first in the total number of citations, with 1282 citations. Of the top 10 institutions, 4 were in the United States, 2 were in China, 2 were in Australia, one was in Germany and one was in Singapore. Those with centrality values greater than 0.1 included the New England College of Optometry, the University of Houston, Fudan University, the Australian National University, the National University of Singapore, and Queensland University of Technology. The Australian National University had the highest centrality (0.19), indicating that it occupies a prominent position in light and myopia research. Figure 3 shows the co-occurrence network of institutional collaborations.

**Table 3:** The top 10 institutions with the highest number of publications.

Rank	Organization	Country	Number of publications	Number of citations	Average citation/publication	Centrality
1	New England College Optometry	USA	55	1151	20.9	0.14
2	University of Houston	USA	45	1167	25.9	0.17
3	Fudan University	China	31	627	20.2	0.12
4	University of Tübingen	Germany	30	1282	42.7	0.06
5	Sun Yat-Sen University	China	30	395	13.2	0.09
6	Australian National University	Australia	25	778	31.1	0.19
7	National University of Singapore	Singapore	23	1212	52.7	0.15
8	Queensland University of Technology	Australia	22	451	20.5	0.14
9	Emory University	USA	21	665	31.7	0.08
10	University of Pennsylvania	USA	18	1037	57.6	0.07

### 3.4. Analysis of authors

**Figure 4.**
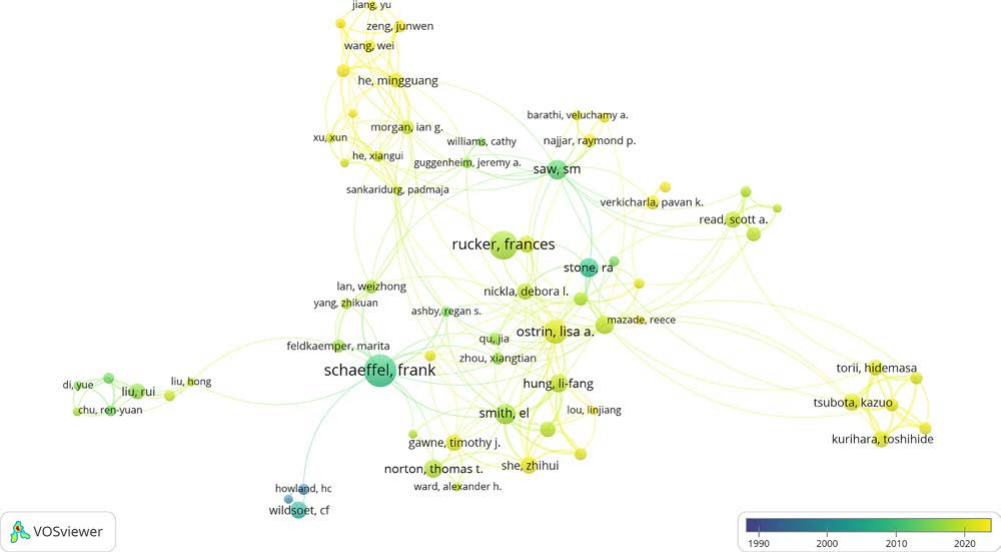
The author research collaboration networks on the relationship between light and myopia. This figure overlays a temporal dimension on the author collaboration network. The colors indicate the period during which scholars were most active. Node size represents the number of publications, and node color represents the time distribution (average year of publication of papers).

A total of 1474 authors have published papers in this field. An analysis of the authors can provide information on the core research strengths in this field of study. According to the aforementioned Price’s Law and Table 4, $n_{max}=42$ and m ≈ 5. Therefore, authors with ≥5 publications were considered core authors in this field of study. From VOSviewer, a total of 69 core authors were identified, and a collaborative network among core authors was generated with a threshold of 5 publications (see Fig. 4). Table 4 shows the top 10 authors with the most publications, with Schaeffel Frank contributing the most publications (n = 42) and receiving the highest number of citations (n = 1939) for light and myopia research. Stone, RA had the most citations per publication (n = 65.1). Among these authors, 8 were from the United States, one was from Germany, and one was from Singapore.

**Table 4:** The top 10 authors with the highest number of publications.

Rank	Author	Country	Organization	Number of publications	Number of citations	Average citation/publication
1	Schaeffel, Frank	Germany	University of Tübingen	42	1939	46.2
2	Rucker, Frances	USA	New England College of Optometry	33	448	13.6
3	Ostrin, Lisa A.	USA	University of Houston	25	460	18.4
4	Smith, Earl L.	USA	University of Houston	19	608	32.0
5	Saw, Seang-Mei	Singapore	National University of Singapore	18	1089	60.5
6	Stone, Richard A.	USA	University of Pennsylvania	17	1107	65.1
7	Pardue, Machelle T.	USA	Emory University	16	654	40.9
8	Norton, Thomas T.	USA	University of Alabama at Birmingham	16	441	27.6
9	Hung, Li-Fang	USA	University of Houston	16	412	25.8
10	Wildsoet, Christine F.	USA	University of California, Berkeley	14	666	47.6

### 3.5. Distribution of journals

These publications were published in 107 journals, which are ranked according to the number of papers published. The top 10 journals are listed in Table 5. The top 3 were *Investigative Ophthalmology and Visual Science, Experimental Eye Research, Vision Research, and Investigative Ophthalmology and Visual Science,* which ranked first in the number of publications (n = 149) and number of citations (n = 3108), placing it far ahead of other journals. *Ophthalmology* had the most citations per article (n = 52.2), indicating that this journal published high-quality articles, followed by *Vision Research* (n = 41.3) and *Experimental Eye Research* (n = 33.4). The journals with the top 3 impact factors were *Ophthalmology* (n = 13.7), *Scientific Reports* (n = 4.6), and *Investigative Ophthalmology and Visual Science* (n = 4.4).

**Table 5:** The top 10 Journals with the highest number of publications.

Rank	Journal	Number of Publications	Number of Citations	Average Citation/Publication	Journal Impact Factor (2022)	Journal Citation Reports (2022)
1	Investigative Ophthalmology & Visual Science	149	3108	20.9	4.4	Q1
2	Experimental Eye Research	51	1701	33.4	3.4	Q2
3	Vision Research	44	1819	41.3	1.8	Q3
4	Optometry And Vision Science	28	439	15.7	1.4	Q4
5	Ophthalmic And Physiological Optics	26	673	25.9	2.9	Q2
6	Plos One	16	480	30.0	3.7	Q2
7	Ophthalmology	12	626	52.2	13.7	Q1
8	British Journal of Ophthalmology	10	210	21.0	4.1	Q1
9	Scientific Reports	10	118	11.8	4.6	Q2
10	Journal of Vision	8	128	16.0	1.8	Q3

### 3.6. Analysis of the 10 most cited articles

As shown in Table 6, the most frequently cited article was written by Saw, SM^[34]^ and was published in 2002, with 323 citations. The second most frequently cited article was written by Wildsoet, CF.^[32]^ The author of the third-ranked article was Guggenheim, JA.^[10]^ Of the ten most cited papers, the earliest was written by Schaeffel, F. and was published in 1991.^[29]^ Notably, Wu, PC’s article, published in 2018,^[35]^ was one of the most frequently cited high-quality studies on the relationship between light and myopia in the past 5 years (n = 206).

**Table 6:** The top 10 most cited articles.

Rank	Document	Year	Author	Journal	Citations	Document Type
1	Nearwork in early-onset myopia	2002	Saw, SM (Singapore)^[3^^4^^]^	Investigative Ophthalmology & Visual Science (Q1)	323	Clinical Research
2	Active emmetropization - evidence for its existence and ramifications for clinical practice	1997	Wildsoet, CF (USA)^[32]^	Ophthalmic and Physiological Optics (Q2)	256	Review
3	Time outdoors and physical activity as predictors of incident myopia in childhood: a prospective cohort study	2012	Guggenheim, JA (UK)^[^^10^^]^	Investigative Ophthalmology & Visual Science (Q1)	253	Clinical Research
4	The effect of ambient illuminance on the development of deprivation myopia in chicks	2009	Ashby, RS (Australia)	Investigative Ophthalmology & Visual Science (Q1)	233	Basic Research
5	An updated view on the role of dopamine in myopia	2013	Feldkaemper, MP (Germany)	Experimental Eye Research (Q2)	220	Review
6	Myopia prevention and outdoor light intensity in a school-based cluster randomized trial	2018	Wu, PC (Taiwan, China)^[^^35^^]^	Ophthalmology (Q1)	206	Clinical Research
7	Time spent in outdoor activities in relation to myopia prevention and control: a meta-analysis and systematic review	2017	Xiong, SY (China)	Acta Ophthalmologica (Q2)	205	Review
8	Time outdoors and the prevention of myopia	2013	French, AN (Australia)	Experimental Eye Research (Q2)	198	Review
9	Properties of the feedback loops controlling eye growth and refractive state in the chicken	1991	Schaeffel, F (Germany)^[29]^	Vision Research (Q3)	192	Basic Research
10	Protective effects of high ambient lighting on the development of form-deprivation myopia in rhesus monkeys	2012	Smith, EL (USA)	Investigative Ophthalmology & Visual Science (Q1)	188	Basic Research

### 3.7. Keyword co-occurrence analysis

**Figure 5.**
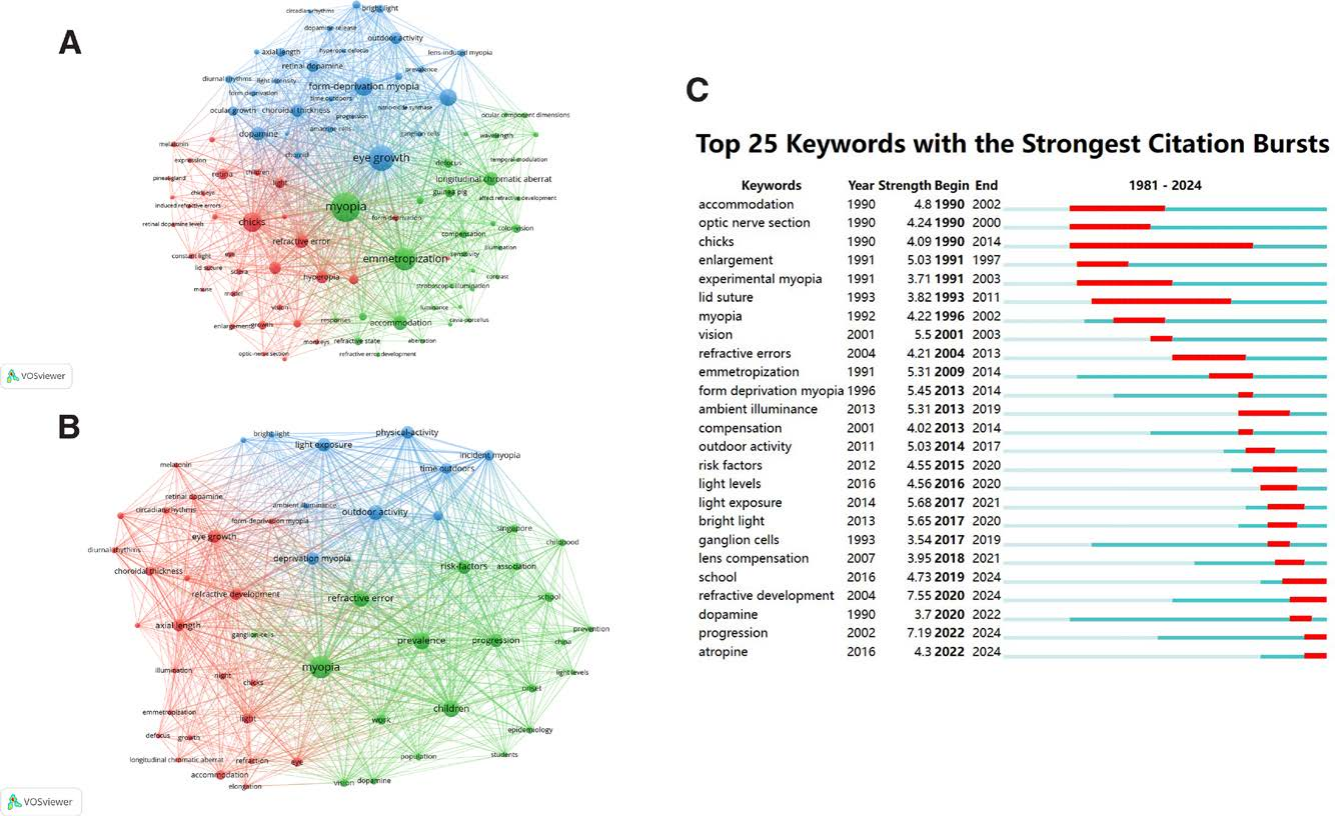
(A) Keyword co-occurrence map of basic research articles. (B) Keyword co-occurrence map of clinical research articles. (C) Top 25 keywords with the strongest citation bursts.

The co-occurrence of keywords in basic research articles is shown in Figure 5A. Cluster 1 (red) represents studies on light and refractive error, and the extracted keywords included “refractive error,” “chicks,” “hyperopia,” “light” and “growth.” Cluster 2 (green) represents studies related to light and emmetropization, and the extracted keywords mainly included “myopia,” “emmetropization,” “longitudinal chromatic aberration,” “compensation,” and “accommodation.” Cluster 3 (blue) represents studies on the relationship between light and induced myopia, and the extracted keywords mainly included “eye growth,” “form-deprivation myopia,” “choroidal thickness,” “dopamine,” and “refractive development.”

The co-occurring keywords used in clinical research are displayed in Figure 5B. Cluster 1 (red) represents studies related to light and refractive development, with the representative keywords “eye growth,” “refractive development,” “axial length,” “choroidal thickness,” and “illumination.” Cluster 2 (green) represents research related to the risk factors for myopia, with the representative keywords “myopia,” “school,” “prevalence,” “children,” and “risk factors.” Cluster 3 (blue) represents light exposure related studies, with the representative keywords “light exposure,” “outdoor activity,” “physical activity,” “ambient illuminance,” and “bright light.”

Figure 5C shows the top 25 keywords with the strongest citation bursts in light and myopia research. “Chicks” lasted the longest, appearing in 1990 and lasting until 2014 (a burst period of 24 years). “Refractive development,” “progression,” and “light exposure” were the 3 keywords with the highest citation strength. Before 2010, the highly cited keywords were “vision,” “enlargement,” and “accommodation,” while after 2010, they were “refractive development,” “progression,” and “light exposure.”

### 3.8. Cocitation analysis

**Figure 6.**
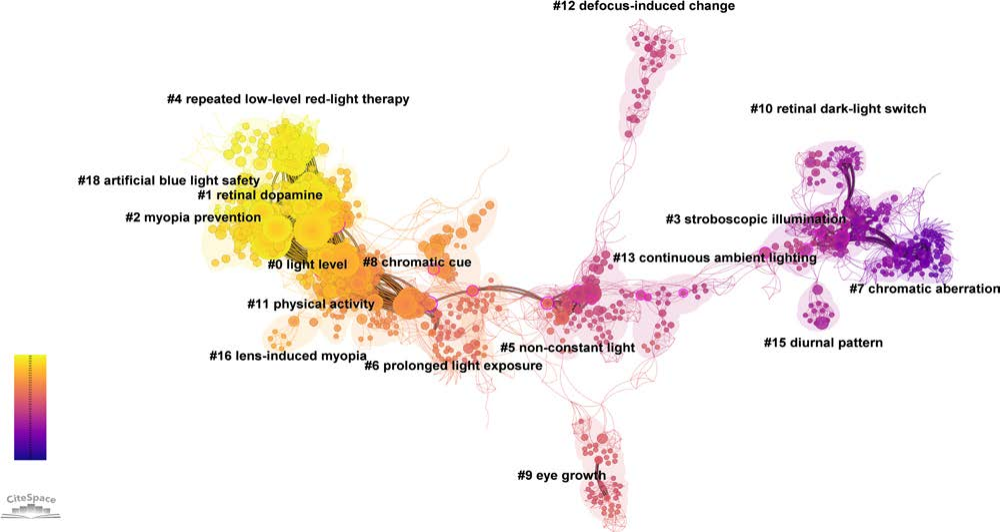
Cocitation clustering analysis of the relationship between light and myopia. Within a cluster, each node represents a cited publication and is connected to other nodes through lines. The size of a node indicates the number of times a publication has been cited, while the color scheme indicates the time span (gradual shift from blue to orange). The deeper the color is, the earlier the publication year.

CiteSpace was used to perform cocitation analysis on 525 publications. As shown in Figure 6, a total of 1094 cocited references were selected for analysis, generating 17 clusters: # 0 light level, #1 retinal dopamine, #2 myopia prevention, #3 stroboscopic illumination, #4 repeated low-level red-light therapy, #5 non-constant light, #6 prolonged light exposure, #7 chromatic aberration, #8 chromatic cue, #9 eye growth, #10 retinal dark-light switch, #11 physical activity, #12 defocus-induced change, #13 continuous ambient lighting, #15 diurnal pattern, #16 lens-induced myopia, and #18 artificial blue light safety.

## 4. Discussion

Myopia has become a prevalent disease, and methods for preventing and slowing its progression are urgently needed. Light exposure has great potential for myopia prevention, but further exploration of this relationship is needed. Bibliometrics can provide a scientometric analysis, summarize a large amount of literature, and elucidate a particular field’s hotspots and research trends. We conducted a detailed bibliometric analysis and visualization of the literature on the relationship between light and myopia published between 1981 and 2024, aiming to identify the hotspots and frontiers of this research field and provide a valuable reference for healthcare professionals.

### 4.1. Research on the relationship between light and myopia

The trends of publication volume reflect the development speed of a research field. The limited publication output in the first stage of this field may be attributed to the insufficient scholarly attention it has received. However, the number of papers in the second stage is significantly higher than that in the first stage, indicating that the field has emerged as a prominent research topic since 2013. Clinical research has become a focal area of investigation within this domain. Notably, the United States not only has the highest contribution of papers to this field but also a majority of the institutions and authors involved are affiliated with the United States, possibly because the United States has many top universities and research institutions with first-class research facilities and resources, providing a superior research environment and conditions for researchers. Singapore has the highest average citation count due to its prestigious reputation in myopia research and the high prevalence of myopia in Southeast Asia. Importantly, 5 of the top 10 countries with the most publications were in Asia, which may be related to the region’s increasing myopia incidence in recent years.^[4]^ As shown in Figure 4, there were several collaborative groups in this study area, but there was not much collaboration between groups. It is hoped that cooperation between authors will increase in the future. *Ophthalmology* had the highest average citation frequency per paper, indicating that the articles published in this journal were of high quality and highly recognized by many researchers.

The most frequently cited article was authored by Saw, SM.^[34]^ This article assessed the relationship between light exposure history and myopia. The results showed that night lighting before age 2 was not significantly associated with the severity of myopia. The second most cited article was written by Wildsoet, CF.^[34]^ This paper proposed that chromatic aberration was involved in the process of emmetropization in chicks. The author of the third-ranked article was Guggenheim, JA.^[10]^ The article claimed that the relationship between outdoor activity and myopia development may be mediated by the levels of natural light outdoors and the spectral composition of ambient light. The earliest paper (published in 1991) investigated whether different wavelengths of light may act as defocusing signals and thus influence the process of emmetropization.^[29]^ In the past 5 years, the article “*Myopia Prevention and Outdoor Light Intensity in a School-Based Cluster Randomized Trial*” by Wu, PC, has been highly cited.^[35]^ The findings of this study indicate that compared to the control group, the intervention group exposed to at least 11 hours of outdoor activity per week (at 1000 lux or higher) exhibited a substantial reduction in myopic shift and elongation as well as a 54% lower risk of rapid myopia progression.^[35]^ This study provides a reference for the use of outdoor light exposure to postpone and prevent myopia. Although ranking by citation number might be biased against recently published studies, 6 of the top 10 articles with the highest citation numbers were published after 2010. This indicates that the relationship between light and myopia has received increasing academic attention in recent years.

Keywords reflect the core content and topic of a paper. The primary keywords for basic research were “refractive error,” “longitudinal chromatic aberration,” “emmetropization,” and “dopamine.” Emmetropization and longitudinal chromatic aberration mechanisms are two fundamental components of myopia formation theories. Some scholars believe that the mechanism by which light inhibits myopia progression is related to the secretion of retinal dopamine. Ashby et al^[33]^ showed that intraocular injection of the D2 dopaminergic antagonist spiperone into chicks eliminated the protective effect of bright light on deprivation myopia. In summary, the above keyword clustering analysis indicated that basic research on light and myopia has focused mainly on the role of light in myopia onset. The main keywords of clinical research included “illumination,” “school,” “outdoor activity,” and “risk-factors.” This indicated that clinical research has focused more on the role of light in the process of myopia prevention and control, especially among school-age children. The evolution of keyword bursts revealed that the focus of attention in the field is currently changing. Before 2010, this field focused on the pathogenesis of the disease, while after 2010, the emphasis shifted toward myopia prevention and control. The emerging keywords “school,” “progression,” and “light exposure” align with the current state where school-aged children and adolescents have become high-risk populations for myopia.

### 4.2. Research trends and hotspots regarding the relationship between light and myopia

Scientific literature is an interconnected system, and mutual citations among scientific publications can reflect a knowledge domain’s structure and dynamic development, providing scholars with a holistic view of the field.^[36]^ Cocitation reflects subject similarity. Small defined it as “the frequency with which two documents were cited together”^[37,38]^; in other words, research clusters form when many authors cite the same two papers in one publication, and the papers in clusters with shared citations typically share a common theme, making each cluster representative of a unique research topic.^[37]^ To delineate different research topics in the field, the clustering function of CiteSpace was employed to partition the entire cocitation analysis into distinct clusters composed of cited studies. The cluster labels in CiteSpace were extracted based on the titles, abstracts, and keywords of the citing publications. Chen suggested that an optimal labeling method could differentiate emerging trends and rapid changes in the foreground from more enduring themes in the background.^[25]^ He also noted that the citing articles represent the research frontiers, and the cited articles represent the intellectual base.^[39]^ Therefore, by utilizing the cocitation function of CiteSpace, it was possible to explore research frontiers or hotspots based on the intellectual base. This study focused mainly on the relationship between light and myopia. The following discussion is divided into several themes based on the cocitation clustering results.

### 4.3. The relationship between the properties of light and myopia

#### 4.3.1. Light levels and myopia

This theme was mainly composed of the following clusters: #0 light level, #1 retinal dopamine, #12 defocus-induced change, and #16 lens-induced myopia.

Light level, or light luminance, has recently been a research hotspot, and Cluster 0 (light level) in Figure 6 was the largest cluster in the citation analysis. In fact, as early as 1886, Cohn suggested that improving school lighting levels could prevent myopia in schoolchildren.^[40]^ Recently, light intensity has again become a research hotspot. Outdoor activity is a protective factor against myopia that has been widely studied in recent years, and the mechanism of this protective effect may be related to the level of outdoor illumination, as there is a large difference in light levels between indoor and outdoor environments.^[41]^ On sunny days, the light level can reach 130,000 lux, which is approximately 10 to 1000 times higher than indoors.^[42]^ Studies have shown that high levels of outdoor light exposure may be related to a reduced incidence of myopia.^[43,44]^

Similar conclusions have also been demonstrated in animal experiments. In chicks exposed to high levels of light (15,000–30,000 lux), the progression of experimentally induced myopia slowed down and the emmetropization process was delayed, leading to more hyperopic refractive errors.^[45,46]^ However, some experimental findings have suggested that the mechanisms underlying form-deprivation myopia (FDM) and lens-induced myopia (LIM) differ. Karouta et al^[47]^ reported that high-intensity light exposure suppressed the development of FDM in chicks, and exposure to light levels of 40,000 lux almost completely prevented the occurrence of FDM. Similarly, in primates, studies have shown that exposure to high-intensity light delays the development of FDM. In contrast, in primates, high light levels did not significantly alter the LIM process and did not change the endpoint of emmetropization, indicating that the compensatory degree of negative lens-induced hyperopic defocus was the same regardless of whether the animals were exposed to high or moderate light. These results suggest that FDM and LIM are fundamentally distinct,^[48]^ at least as observed in primate models. However, some researchers have suggested that the effects of high light levels may vary according to species. For example, high light levels can delay the development of negative lens-induced myopia in chicks but have little effect on primates.^[45]^ While some of these experiments have supported the hypothesis that high-level light exposure is a protective factor against myopia, it remains a mystery whether the results of animal experiments can be extrapolated to humans.

Historically, clinical studies have relied on questionnaire surveys to collect data. However, a study conducted by Wu PC employed wearable devices to intensively sample individual light exposure data, aiming to obtain detailed and objective information. This method effectively mitigated subjective biases.^[49,50]^ The results of this study indicate that short periods of high light intensity or longer exposure to moderate light intensity effectively reduce myopia in both nonmyopic and myopic children.^[35]^ Notably, these findings differ from those observed in animal experiments, where only prolonged exposure to high light intensity was found to delay experiment-induced myopia. Nonetheless, Wu’s study presents an alternative approach to investigating light levels and highlights the potential effectiveness of moderate light levels in preventing myopia. Importantly, although high-intensity light may be useful in preventing myopia, it may also result in other health issues, such as skin cancer, which should not be overlooked.

As the prevailing hypothesis in the field, the dopamine hypothesis has garnered substantial support from numerous experimental studies. This hypothesis proposes that bright light suppresses the progression of myopia by promoting the synthesis and secretion of dopamine in the retina. For instance, in animal experiments involving interventions to induce experimental myopia in rhesus monkeys, dopamine levels were significantly lower in the experimental group than in the control group.^[51]^ Some experimental results suggest that the inhibitory effects of dopamine agonists on FDM and LIM are mediated by the stimulation of D2-like receptors. Additionally, the sole use of D2 receptor antagonists does not affect normal eye growth.^[52,53]^ Another in vitro study revealed that increasing light intensity can elevate the mRNA levels of D1 and D5 receptors, indicating the potential involvement of other receptors and suggesting that different dopamine receptors may play distinct roles in this process.

In addition, another hypothesis suggests that exposure to bright outdoor light may protect against myopia because the outdoor environment is a three-dimensional structure, and light may influence the mechanisms responsible for myopic and hyperopic compensation, thereby changing the dynamics of the responses to myopic and hyperopic defocus.^[54]^ This finding implies that the relationship between hyperopic and myopic defocus may differ between outdoors and indoors. Furthermore, the greater uniformity of the refractive structure of the outdoor environment may also be an important factor. In the future, researchers may further explore the effective range of light levels, specific molecular pathways mediated by dopamine receptors, and the relationship between outdoor environments and bright light exposure.

#### 4.3.2. Light wavelengths and myopia

This theme was mainly composed of the following clusters: #4 repeated low-level red-light therapy, #7 chromatic aberration, #8 chromatic cue, and #18 artificial blue light safety.

Emmetropization is the process by which the eyes adjust their growth rate by recognizing varying defocused signals projected onto the retina, thereby ensuring a match with optical power and reducing refractive errors.^[55]^ Longitudinal chromatic aberration (LCA) is an optical concept that refers to the phenomenon in which light of different wavelengths is refracted by an optical system and focused at different positions. In 1947, Wald et al conducted the first assessment of LCA and reported a 1.75 diopter chromatic difference between 420 and 660 nm.^[56]^ Similarly, Charman,^[55]^ posited that there is an approximately 2 diopter difference in focus across the visible spectrum, which can result in color blur. Emmetropization utilizes this color blur as a signal to guide the growth of the eye.

LCA transmits a wavelength defocus signal and a chromatic signal of broadband light; however, for narrowband monochromatic light, LCA transmits only a wavelength defocus signal.^[57]^ Both wavelength defocus signals and chromatic signals have an impact on emmetropization. The wavelength defocus signal guides emmetropization through the wavelength. The chromatic signal guides emmetropization by comparing relative cone contrast.^[58]^ For instance, rearing chicks, rainbow trout, and guinea pigs under red light has been shown to increase their susceptibility to myopia; the growth of the chick eye will increase when the contrast of short-wavelength-sensitive cones exceeds that of long-wavelength-sensitive cones and middle-wavelength-sensitive cones.^[59–61]^ However, young and adolescent tree shrews exposed to red light for 13 days exhibit significant hyperopia, possibly because long wavelengths are focused while short wavelengths are not, which may indicate that the eye has become long enough and should slow the rate of axial elongation.^[62,63]^ The above results indicate that the LCA mechanism may use narrowband wavelength information to control the eye growth rate to match the focal plane of specific wavelengths. Similarly, the LCA mechanism can also serve as a cue, indicating that long-wavelength light focuses better than short-wavelength light. The reasons for this phenomenon are currently unclear and may be related to luminous intensity, age, species, specific wavelength distributions, and the number of cone types, among other factors^[64]^; further research is needed.

In addition to these animal experiments, some clinical studies have shown that red light can be used to treat myopia. A multicenter, randomized, parallel, single-blind clinical trial from China showed that repeated low-level red light therapy for 12 months inhibited axial elongation and myopia progression by 69.4%, and was more effective than wearing OK lenses, indicating that repeated low-level red light therapy is a promising alternative therapy.^[65]^ However, the mechanism by which red light therapy can treat myopia remains unclear and may be related to the abundance of mitochondria in photoreceptor cells in the eye. This contradicts the classic emmetropization feedback theory.^[66]^ Despite promising results in treating and slowing myopia, the safety of red light therapy has been controversial. Some researchers have noted the potential risks of retinal photochemical and thermal damage associated with red light.^[31]^ Therefore, careful consideration of the potential risks and benefits is warranted before considering the adoption of red light therapy for myopia.

Unlike red light, blue light is a short-wavelength, high-energy light with a wavelength between 415 and 455 nm. Rucker et al suggested that exposure to natural light with a higher content of short-wavelength light can slow axial growth.^[30]^ Additionally, animal experiments have shown that blue light can inhibit the axial length growth of guinea pigs and induce hyperopia.^[60]^ Some researchers believe that blue light affects refractive development by inducing changes in cone cell density. However, blue light may induce the production of a considerable number of free radicals, disrupting the body’s normal redox state and consequently damaging the cornea, lens, and retina.^[67]^ Therefore, ignoring the possible damage caused by blue light when considering its advantages is not advisable.

Violet light (VL) is the shortest wavelength light in the visible spectrum, with a wavelength range of 360 to 400 nm. Jiang et al^[68]^ demonstrated that violet light prevented defocus-induced myopia in mice, and the protective effect of violet light against myopia may be related to a photopigment called neuropsin. Upregulation of the expression of early growth response protein 1, a myopia-protective gene, is also one of the mechanisms by which violet light prevents myopia.^[69]^ A retrospective clinical trial conducted by Torii et al^[70]^ demonstrated that, compared to individuals in the control group, individuals with high myopia who underwent implantation of phakic intraocular lenses with VL-transmitting capabilities showed a significant reduction in the progression of refractive error and axial length after 5 years. These results suggest that VL may play a role in preventing and controlling myopia. Because the wavelength of VL is less than 360 nm, VL is rarely detected in indoor environments, which may be one of the reasons contributing to the increasing prevalence of myopia worldwide. However, it cannot penetrate the cornea or lens, rendering it a potentially safe and ideal wavelength for future myopia prevention and control.^[71]^

The above results indicated that the influence of LCA on emmetropization varied depending on the species and experimental conditions. Despite these differences, the evidence suggested that the development of refractive function was actively regulated by visual feedback generated by the effective refractive state of the eye (wavelength defocus signal, chromatic signal). The spectral composition might influence the emmetropization process driven by optical defocusing in various ways, thus affecting the development of ocular refractive function.

#### 4.3.3. Other features of light related to myopia

This theme mainly comprised the following clusters: #3 stroboscopic illumination, #5 nonconstant light, #6 prolonged light exposure, #10 retinal dark-light switch, #13 continuous ambient lighting, and #15 diurnal pattern.

Other features of light also have varying degrees of impact on refractive development, such as the frequency and rhythm of light exposure. Some researchers have suggested that defocus-induced myopia and deprivation-induced myopia can both be suppressed by stroboscopic light.^[72,73]^ According to Di et al, myopia can be induced in guinea pigs through exposure to illumination with a duty cycle of 50% at a flash rate of 0.5 Hz. Other studies have shown that exposure to light flickering at frequencies above 6 Hz inhibits myopia induced by either deprivation or hyperopic defocus in species such as chickens, while lower-frequency flickering, ranging from 1 to 4 Hz, has been shown to aggravate experimental myopia.^[74]^ Comparable trends have been observed in guinea pigs, cats, and mice, where stimuli with high contrast and high frequencies (10 Hz) have been found to reduce eye growth, while low frequencies (0.2–2 Hz) have been shown to increase eye growth. This may be due to histological damage to the retina induced by prolonged exposure to low-frequency modulated light and the resultant exaggerated axial elongation of the eye.^[75]^ When exposed to low-frequency modulated light, the retinal image becomes incoherent, generating less neural activity and leading to remodeling of the sclera, which in turn causes axial elongation of the eye.^[76]^ In summary, these differences may be attributed to species-specific factors as well as the frequency and chromatic contrast of the flickering stimuli, among other factors, resulting from complex interactions among multiple factors.

Lighting rhythms can also affect refractive development, resulting in myopic outcomes, such as ambient lighting or nighttime illumination fluctuations. Quinn et al conducted a survey-based study and observed strong associations between the prevalence of myopia and high myopia during childhood with ambient light exposure during sleep at night in the first 2 years after birth. The authors suggested that the lack of a daily period of darkness could precipitate the subsequent development of myopia.^[77]^ The underlying mechanism responsible for the effect of ambient lighting during sleep on refractive development is primarily the function of the human eyelid as a red-pass filter. The eyelids of both human infants and adults permit the transmission of specific long-wavelength light.^[78,79]^ Hence, it is reasonable to speculate that these long-wavelength lights could contribute to developing myopia. However, other survey-based studies have reported no significant difference in the prevalence of myopia between children who slept in the dark before the age of 2 and those who were exposed to ambient light at night.^[34,80]^

Seasonal changes in lighting may also affect refractive development. In 1972, Lythgoe demonstrated that twilight and night skies tend to have more red light than bright daylight.^[81]^ Decades later, Rodieck reported that the light/dark cycle could impact refractive development.^[82]^ In 2003, Vannas observed an increased prevalence of myopia in those living in the Arctic Circle, likely due to the extreme seasonal changes in lighting in Finland’s unique geographical location.^[83]^ Although the results of these studies are not entirely consistent, they provide new insights into the mechanisms underlying myopia and effective prevention measures.

### 4.4. The relationship between light and myopia prevention

This theme mainly comprised the following clusters: #2 myopia prevention, #9 eye growth, and #11 physical activity.

Myopia is incurable; therefore, the importance of preventing its occurrence far outweighs that of treating myopia. Among the numerous methods for myopia prevention and control, increasing outdoor activities is a cost-effective and efficient approach. Multiple studies have shown that outdoor activities can inhibit the progression of myopia, and children who spend more time outdoors are less likely to develop myopia.^[84–86]^ Wu^[86]^ showed that the intervention group (with outdoor activities) had a 9% reduction in the incidence of myopia compared to the control group. In 2007, Jones et al reported that less physical activity and less time spent outdoors increased the risk of myopia in children whose parents were myopic.^[8]^ In 2008, Rose et al^[43]^ demonstrated that outdoor activities reduced the prevalence of myopia in children. Recently, several researchers have proposed that the different spatial frequency compositions of indoor and outdoor environments are potential mechanisms leading to myopia and that manipulating the visual environment of young people may be helpful in preventing or inhibiting the progression of myopia.^[74,87]^ To date, a crucial question that requires further investigation is whether the protective effect of outdoor time against myopia is primarily mediated by the intensity and/or duration of light exposure or by other environmental factors associated with outdoor activity. Furthermore, despite identifying numerous risk factors for myopia, only a limited number of randomized controlled trials have demonstrated that interventions are effective for myopia prevention and control. The scarcity of randomized controlled trials in myopia research may be attributed to ethical considerations arising from the potential adverse effects of certain interventions on children’s physical and psychological well-being. In summary, a more comprehensive and systematic evaluation of myopia prevention and control strategies is necessary for their successful implementation in clinical practice.

## 5. Conclusion

This study is the first to apply bibliometric analysis to investigate the relationship between light and myopia. This review revealed the knowledge structure, research hotspots, and future developmental trends in the field based on studies published between 1981 and 2024, providing valuable information for researchers to explore further.

The adoption of “Topic” as the retrieval strategy in this study is motivated by several factors. First, during the initial stages of this research, attempts to utilize “Titles” as search criteria resulted in an insufficient number of target documents, leading to the omission of certain literature. Using “Title” search criteria imposes stricter limitations, thereby enhancing precision but potentially sacrificing comprehensiveness. In contrast, “Topic” search criteria offer broader parameters, resulting in a more comprehensive scope, albeit with decreased precision. Second, Jie Li and Chaomei Chen recommend using broader “Topic” searches when conducting CiteSpace analysis on retrieval data. Based on these considerations, “Topic” was employed as the retrieval strategy for this study. In the later stages of data cleaning, meticulous scrutiny was applied to the “titles,” “abstracts,” and “keywords” of the literature to ensure high precision.^[88]^

However, this study had several limitations. First, only the Web of Science Core Collection database was used, and articles not included in this database could not be analyzed; therefore, future studies should consider the use of additional databases. Second, this study was limited to studies published in English, which may introduce language bias. Overall, this study provides direction for future research in this field. The close association between light and myopia highlights the potential of utilizing light to slow or prevent myopia progression, informing public health strategies and interventions. Moreover, light exposure is cost-effective and environmentally friendly, making it a highly promising tool for myopia prevention and control in the future.

## Author contributions

**Conceptualization:** Hongmin Zhang.

**Data curation:** Shuaibing Zhou, Yueyue Niu.

**Formal analysis:** Shuaibing Zhou, Yueyue Niu.

**Methodology:** Shuaibing Zhou.

**Software:** Shuaibing Zhou.

**Supervision:** Juan Yue, Hongmin Zhang.

**Validation:** Xuejiao Li, Hongmin Zhang.

**Writing – original draft:** Shuaibing Zhou, Yueyue Niu.

**Writing – review & editing:** Xuejiao Li, Juan Yue, Hongmin Zhang.
